# Singlet Oxygen Generation and Signaling in Higher Plants

**DOI:** 10.3390/ijms27031462

**Published:** 2026-02-01

**Authors:** Huan Zhao, Xinyue Wang, Liangsheng Wang

**Affiliations:** 1 State Key Laboratory of Plant Environmental Resilience, College of Biological Sciences, China Agricultural University, Beijing 100193, China; 15652519898@163.com (H.Z.); wxy131234@163.com (X.W.); 2 Frontiers Science Center for Molecular Design Breeding (MOE), China Agricultural University, Beijing 100193, China

**Keywords:** chloroplast, singlet oxygen, signaling, stress, phytohormone

## Abstract

Singlet oxygen (^1^O_2_), the excitation stage of the ground-state molecular oxygen, is a fundamental reactive oxygen species (ROS) with important functions in plant growth, development, and stress responses. In plant cells, ^1^O_2_ is mainly generated in the chloroplast due to photosensitizing activity of tetrapyrroles. Moreover, ^1^O_2_ can be generated in non-photosynthetic tissues when plants suffer environmental stresses. Although ^1^O_2_ was initially considered as a cytotoxin—causing merely photooxidative damages, more recent work suggests that ^1^O_2_ also acts as a signal that either triggers a programmed cell death response or promotes acclimation. The ^1^O_2_ signaling pathway is distinct and operates independently of other ROS signaling cascades. In Arabidopsis, EXECUTER1 (EX1) protein has been identified as a crucial signaling component that perceives and relays ^1^O_2_ signals to the nucleus, thereby initiating extensive transcriptional reprogramming. Additionally, oxidative products of carotenoids, such as β-cyclocitral, are also recognized as ^1^O_2_-derived signaling molecules. Through specific chloroplast-to-nucleus signaling and cross talk with hormone signaling networks—including jasmonic acid (JA) and salicylic acid (SA)—^1^O_2_ helps finely coordinate plant growth, defense responses, and cell fate decisions under fluctuating environmental conditions. This review aims to summarize current knowledge on ^1^O_2_ generation and signaling, ^1^O_2_-induced chloroplast changes under diverse stress conditions, and cross talk between ^1^O_2_ and phytohormone signaling.

## 1. Introduction

Plants performing oxygenic photosynthesis inevitably generate a certain amount of ROS. While a basal level of ROS is necessary for various metabolic processes, a sudden increase in ROS is detrimental to plants [1]. Under stress conditions such as drought, high temperature, and high light that interfere with photosynthetic energy or electron transportation, a large amount of ROS including hydrogen peroxide (H_2_O_2_), singlet oxygen (^1^O_2_), superoxide radical ($O_{2}^{\cdot -}$), and hydroxyl radical (OH^•^) are generated in the chloroplast [2,3]. Although many kinds of ROS are generated simultaneously, ^1^O_2_ has been proved the most detrimental to the chloroplast as more than 80% of photooxidative damages are caused by it directly [4]. Singlet oxygen is the excitation stage of the ground-state triplet molecular oxygen [5,6,7]. Energy transfer from an excited photosensitizer (e.g., ^3^Chl) could reverse the spin direction of one of the two outermost valence electrons of the triplet-state oxygen that occupy separate orbitals with parallel spins, allowing the pairing of the two electrons and formation of ^1^O_2_ [7]. Singlet oxygen could rapidly oxidize proteins, lipids, carbohydrates, and nucleic acids, and in this way it may irreversibly inactivate/destroy the targets [8,9,10]. Thus, for a long time, ^1^O_2_ had been considered as a cytotoxin because of its extreme high reactivity and short lifetime—about 1 µs in living cells [11,12,13]. However, an increasing body of recent studies clearly demonstrate that ^1^O_2_, in most cases, functions as a versatile signal that induces various stress responses via activating sequential signaling transduction cascades [14,15,16,17,18]. In the review, we summarize current understanding of ^1^O_2_ generation, perception, and signaling and discuss the role of ^1^O_2_ in plant environmental resilience.

## 2. Generation of Singlet Oxygen

In plant cells, ^1^O_2_ is mainly generated in the chloroplast by transferring the excitation energy to the ground-state triplet molecular oxygen (^3^O_2_) from a photosensitizer either metabolically or photochemically. Within the chloroplast, ^1^O_2_ is mainly generated during photosynthesis and from intermediate products of chlorophyll biosynthesis and degradation [19].

### 2.1. Singlet Oxygen Generation During Photosynthesis

In the light-harvesting antenna complex (LHC), chlorophyll (Chl) is excited from the ground state to its singlet excited state (^1^Chl*) after absorbing a photon, and the fate of its excitation energy is different [20,21]. At normal growth conditions, most (80~85%) of the excitation energy is gradually transferred to the reaction center of PSII to drive the photosynthetic electron transfer chain, and the rest of the light energy will be dissipated in the form of heat or fluorescence, a process known as non-photochemical quenching (NPQ) [22,23]. ^1^Chl * is a rather short-lived (about 10^−8^ s in living cells) molecule [20]. If the excitation energy in ^1^Chl * is not immediately used, ^1^Chl * will release a small portion of energy, decaying to its longer-lifespan (about 10^−3^ s) triplet excitation state, ^3^Chl*, via charge recombination [24]. ^3^Chl * is a strong photosensitizer that can transfer its excitation energy to the ground-state oxygen, leading to the generation of ^1^O_2_ [25,26,27].

Though ^3^Chl * is produced, generation of ^1^O_2_ in LHC is rather limited. In the antenna, chlorophylls are surrounded by carotenoids, such as lutein and zeaxanthin, and the generated ^3^Chl * is mostly (about 95%) quenched by the carotenoid molecules presented nearby. Thus, only a trace amount of ^1^O_2_ is produced in LHC, even for plants that are grown under high light [28,29,30,31]. The Arabidopsis *ch1* mutant is devoid of chlorophyll *b* (Chl *b*) due to the inactivation of chlorophyll *a* oxygenase (CAO) that catalyzes the conversion of Chl *a* to Chl *b* [17,32]. Since Chl *b* is an essential component of the light-harvesting complex associated with PSII, the absence of Chl *b* disrupts the formation and stability of the PSII antenna complex, which in turn impairs excitation energy transfer within LHC and leads to enhanced ^1^O_2_ production under high light [33,34,35].

In the PSII reaction center (RC), it is a rather different scenario as a large amount of ^1^O_2_ is produced when plants suffer from high light [36,37]. The PSII RC contains six chlorophyll *a* (Chl *a*) molecules, two pheophytin (Pheo), two β-carotenes (Car), and two quinones arranged along the D1 and D2 branches [38]. Among the six Chl *a* molecules, two of the four Chl *a* molecules located in the center are specialized and paired as primary electron donor, P_680_ [39]. Once it absorbs light energy, P_680_ is excited to its singlet excitation state, ^1^P_680_ *, releasing an energetic electron that is transferred to Pheo and quinone A (Q_A_) sequentially [40,41]. Under stress conditions, it frequently happens that the electron acceptor Q_A_ remains reduced due to the blocking of forward electron transfer (also called the closed state of the reaction center), making it unable to accept electrons [2,42]. In such a condition, the excited ^1^P_680_ * could decay to the long-lived triplet excited state ^3^P_680_ * via the formation of two intermediates, ^1^P_680_^+^Pheo^−^ and ^3^P_680_^+^Pheo^−^ [2,20]. Unlike LHC, P_680_ in PSII RC is not surrounded by carotenoids. The two β-carotene molecules in PSII RC are too far way to quench ^3^P_680_ * directly, that eventually leads to the production of ^1^O_2_ [25,27,43,44,45].

Plastoquinone (PQ) acts as an electron carrier between PSII and Photosystem I (PSI) during photosynthesis, existing in interconvertible oxidized and reduced forms [46]. Excitation of PSII enhances the electron transport rate, leading to the reduction in the PQ pool. In addition to being an electron carrier, PQ also acts as a scavenger of ^1^O_2_ [47,48]. Two plastoglobuli-localized kinases, ABC1K1 and ABC1K3, play a crucial role in maintaining the redox balance of the PQ pool. In the *abc1k1* mutant, the diminished PQ pool compromises photosynthetic electron flow due to insufficient electron acceptors in PSII, and may also impair its capacity to scavenge ^1^O_2_. The *abc1k1* mutant exhibits distinct photosynthetic and metabolic phenotypes—the contents of PQ, β-carotene, and xanthophyll lutein are reduced, and the metabolic process of the membrane antioxidant tocopherol is disrupted [49]. Consequently, the reduction in the PQ pool of the *abc1k1* mutant results in excessive accumulation of ^1^O_2_ and severe cell death phenotypes when grown under red or white light [46].

### 2.2. Singlet Oxygen Generation from Chlorophyll Biosynthesis Intermediates

**Figure 1:**
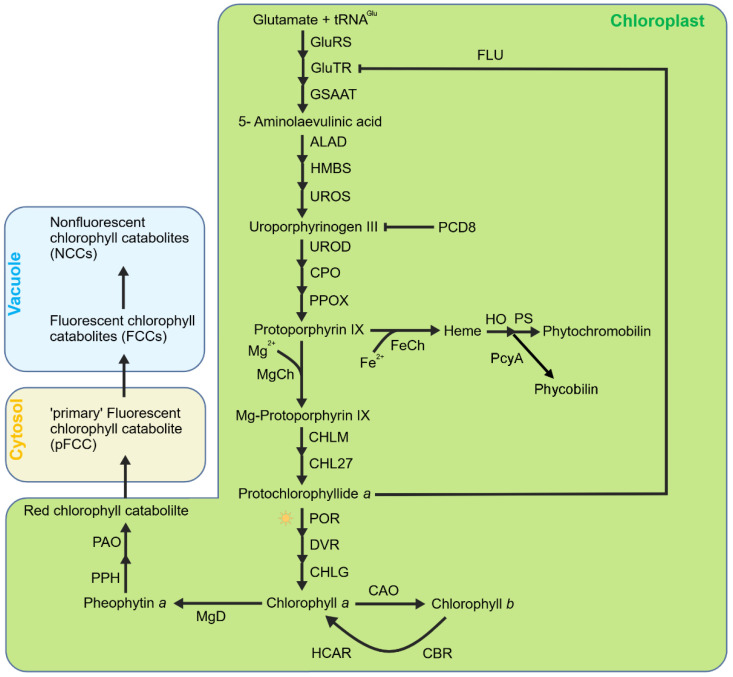
**Simplified model of tetrapyrrole metabolism in chloroplasts.** Tetrapyrrole biosynthesis originates from glutamic acid and shares a common biosynthetic pathway until the formation of protoporphyrin IX (Proto IX). Then, this pathway diverges to the Mg branch (leading to the formation of Chls) and Fe branch (leading to the formation of heme and the subsequent phytochromobilin or phycobilin). Mg–protoporphyrin is subsequently converted to chlorophyll *a*, which can be further converted to chlorophyll *b* by CAO. Chlorophyll degradation begins with the conversion of chlorophyll *a* to pheophytin *a* by releasing the Mg^2+^ ion via MgD (Mg–dechelatase), followed by cleavage by PPH and PAO to produce red chlorophyll catabolite (RCC) and subsequently fluorescent chlorophyll catabolites (FCCs), which are finally converted to non-fluorescent chlorophyll catabolites (NCCs). Heme is the precursor of phytochromoblins and phycobilins.

Another important source of ^1^O_2_ in the chloroplast is the biosynthesis intermediates of tetrapyrroles. In higher plants, tetrapyrroles are mainly divided into three categories, chlorophyll, heme, and phycobilin (Figure 1). Tetrapyrrole biosynthesis originates from glutamic acid and shares a common biosynthetic pathway until the formation of protoporphyrin IX (Proto IX) [50,51]. Afterwards, the pathway diverges into two branches, with the Mg branch leading the formation of chlorophylls and the Fe branch the formation of hemes and phycobilins [52,53]. The first step of the Mg branch is catalyzed by magnesium chelatase (MgCh) to insert an Mg^2+^ ion into Proto IX, forming Mg-Proto IX. Mg-Proto IX was then catalyzed by Mg-Proto IX methyltransferase (MgMT/CHLM) and Mg-Proto IX monomethylester cyclase (MgCY/CHL27) continuously to form protochlorophyllide *a* (Pchlide) in the dark [54,55]. The Mg branch stops when the Pchlide amount reaches a threshold level and restarts when Pchlide is reduced to chlorophyllide *a* (Chlide) by the Pchlide oxidoreductase (POR) under light. The resulting divinyl Chlide *a* is esterified with a long chain polyisoprenyl (geranylgeraniol or phytol) to synthesize Chl *a* [56]. The Fe branch is firstly catalyzed by ferrochelatase (FeCh) to insert Fe^2+^ into Proto IX to form heme *b* which is then converted to other hemes or eventually to phytochromobilins or phycobilins [53].

Free chlorophylls, hemes, and phycobilins are strong photosensitizers due to their structural and biophysical properties [57]. In plant cells, these tetrapyrroles are usually bound with proteins or surrounded by carotenoids to dissipate excess absorbed light energy using various quenching mechanisms. However, the biosynthetic intermediates of tetrapyrroles mostly occurred in free form and are thus more detrimental when illuminated. To prevent overaccumulation, plants have evolved sophisticated strategies to exert strict regulations on tetrapyrrole biosynthesis and degradation, and the most important regulation is the negative feedback inhibition of the initial step of tetrapyrrole biosynthesis, i.e., the formation of δ-aminolevulinic acid (ALA) by two effector molecules, heme and the FLU protein, that both interact with glutamyl-tRNA reductase (Glu-TR), the first enzyme of tetrapyrrole biosynthesis [58,59]. Heme has been implicated in inhibiting mainly the Fe branch [60]. Increased levels of heme bind to the N-terminal part of Glu-TR that blocks Glu-TR activity and inhibits ALA synthesis [60,61,62]. FLU seems to selectively affect the Mg branch of tetrapyrrole biosynthesis [57]. In the dark, Chl biosynthesis ends with the formation of Pchlide. When Pchlide level reaches a critical level, the synthesis of ALA turns down through a yet unknown mechanism that relies on the FLU protein [61,62,63]. The Arabidopsis *flu* mutant, lacking this negative feedback regulation mechanism, is unable to restrain Pchlide accumulation in the dark and thus accumulates an excessive amount of Pchlide [57,61,64]. Once exposed to light, excessive Pchlide transfers light energy to the ground molecular oxygen to produce a large amount of ^1^O_2_, which leads to plant growth inhibition and even cell death [65,66,67]. However, in the *flu* mutant, the heme content is similar to that of wild-type plants, indicating that FLU acts independently of heme [57].

Two ferrochelatases (FeCh), FC1 and FC2, catalyze the formation of heme by inserting Fe^2+^ into protoporphyrin IX [68]. In *fc1* knockout mutants, the level of heme is only slightly decreased in shoot and root, as well as the contents of chlorophylls and carotenoids, and the efficiency of PSII is basically unaffected, indicating that FC1 does not play a major role in tetrapyrrole biosynthesis [69]. However, the *fc2* mutant forms abnormally small, pale green rosette leaves, and is low in chlorophylls and carotenoids, underscoring its major role in tetrapyrrole metabolism [68]. The *fc2* mutant produces an overdose amount of ^1^O_2_ when grown under dark/light cycles that leads to cell death of young seedlings and growth inhibition of mature plants [70,71,72]. However, the underlying reason is still under debate. Woodson et al. reported that the *fc2* mutants accumulate Proto IX after dawn [18], but Scharfenberg et al. pointed that this mutant does not accumulate Proto IX but Pchlide instead in the dark [68].

Homeostasis of tetrapyrrole is important for plants’ developmental programs and plants’ response to environmental cues. Impaired tetrapyrrole biosynthesis or catabolism leads to the generation of ^1^O_2_ and the following cell death responses. PROGRAMMED CELL DEATH 8 (PCD8) is a newly identified thylakoid-localized protein that controls proteolysis of tetrapyrrole biosynthesis proteins by directing Clp protease to these enzymes [73]. Knockdown of the *PCD8* gene leads to the accumulation of ALA, uroporphyrinogen III (Uro III), Proto IX, Mg–protoporphyrin IX (MgP), and Pchlide, resulting in severe chloroplast damage and a necrotic phenotype under short-day conditions. It is proposed that the burst of ^1^O_2_, triggered by excessive accumulation of tetrapyrrole intermediates—particularly uroporphyrinogen III under light conditions—is responsible for the observed cell death in these knockdown mutants [73].

### 2.3. Singlet Oxygen Generation from Chlorophyll Breakdown Products

Another important source of ^1^O_2_ comes from the breakdown process of Chls, especially during senescence. In plant tissues, Chls are usually bound with proteins and carotenoids, and, in this way, the excitation energy absorbed by Chls are readily used for photosynthesis or dissipated as heat or fluorescence [74]. During senescence, Chls are frequently disassociated from these Chl-binding proteins, and the removal of free Chls and its breakdown intermediates is crucial since these molecules readily generate ^1^O_2_ and are potentially toxic to plants [12,75]. Within plastids, a series of enzymes catalyze the conversion of Chl to linear colorless tetrapyrrole derivatives referred to as ‘primary’ fluorescence Chl catabolites (pFCC). pFCC molecules are then exported to the vacuole and finally breakdown to monopyrrole molecules which might be further metabolized to smaller molecules [76,77,78]. Among the Chl breakdown process, the opening of the tetrapyrrole ring that forms the red chlorophyll catabolite (RCC) is recognized as a crucial step, and this step is catalyzed by phenylalanine oxygenase (PAO) [79]. The Arabidopsis *acd1* mutant and maize *lls1* mutant, lacking functional PAO protein, accumulate pheophorbide *a* in the plastids and generate ^1^O_2_ when illuminated [79]. Then RCC is reduced to a ‘primary’ fluorescent chlorophyll catabolite (pFCC) via RCC reductase (RCCR) [80]. The Arabidopsis *acd2* mutant, bearing mutation in the *RCCR* gene, is defective in converting RCC to pFCC, and thus accumulates RCCs and RCC-like pigments in the dark, favoring ^1^O_2_ production when transferred to the light and causing cell death [71,81,82].

### 2.4. Singlet Oxygen Generation During Seedling De-Etiolation

When seeds are geminated and grown in complete darkness, etioplasts are developed in plant tissues that would have chloroplasts if subjected to light. Etioplasts do not contain chlorophyll or stacked thylakoid membranes, but rather have a paracrystalline lipid–pigment–protein structure known as the prolamellar body (PLB) [83]. The PLB consists largely of plastid lipids (mainly MGDG and DGDG), the chlorophyll precursor Pchlide, the light-dependent POR, and its cofactor NADPH [84]. However, if the darkness continues, the level of Pchlide will increase and lead to ^1^O_2_ production after illumination [85]. In etiolated seedlings, the expression of genes required for carotenoid (e.g., *AtPSY*) and chlorophyll biosynthesis (e.g., *AtPOR*) is repressed by the PIF transcription factors [86,87]. Deficiency in either *PIF1* or *PIF3* leads to elevated levels of Pchlide in darkness and results in photobleaching upon transfer to light [86,88]. Upon light exposure, the etiolated *pif5* seedlings also develop a photobleaching phenotype [89]. The photobleaching phenotype observed in seedlings during the dark-to-light transition is largely attributed to ROS [90]. Therefore, researchers observed that, following 24 h of light treatment, 4-day-old dark-grown seedlings of the *pif1 pif3* double mutant and the *pifq* (quadruple) mutant exhibited increased fluorescence from H_2_DCFDA—a dye sensitive to reactive oxygen species—as well as detectable ^1^O_2_ production, as measured by the singlet oxygen sensor green (SOSG) fluorescent probe. These findings indicate that PIFs play a significant role in suppressing ^1^O_2_ generation during seedling de-etiolation [91,92].

### 2.5. Singlet Oxygen Generation in Non-Photosynthetic Conditions

Recent studies indicate that ^1^O_2_ can be generated through multiple stress pathways and may originate from organelles other than chloroplasts in a light-independent manner [93,94,95]. Transcriptional analysis of leaves subjected to various stresses, including drought, reveals ^1^O_2_-induced responses under both dark and light conditions [96]. Researchers observed that the transcriptomic profiles under various stress conditions—whether induced by light or under dark treatments—closely resembled the transcriptome of the *flu* mutant in Arabidopsis [94]. When Arabidopsis roots are subjected to diverse biotic and abiotic stresses in darkness, ^1^O_2_ is rapidly produced and accumulated in mitochondria, peroxisomes, and nuclei. These findings demonstrate that ^1^O_2_ can be produced via multiple stress pathways and may originate from non-chloroplastic organelles without the need for light [94].

Under drought stress, plants exhibit a high osmotic potential. A research team reported that the effect of polyethylene glycol on root growth is strongly correlated with the generation of ^1^O_2_. Once ^1^O_2_ is produced, cell death occurs [97]. Similarly, simulating drought stress through leaf dehydration treatment can rapidly induce ^1^O_2_ production. This result was quantified by measuring the amounts of free radicals captured with the ^1^O_2_-specific spin-trapping agent 4-hydroxy-tetramethylpiperidine [98]. Another study also showed that ^1^O_2_ generates in the wounded Arabidopsis leaves. In the Arabidopsis *lox2* mutant lacking the functional chloroplast lipoxygenase, the production of ^1^O_2_ was suppressed. This effect is due to the absence of lipoxygenase-initiated lipid peroxidation, indicating that chloroplast-localized lipoxygenase plays a role in ^1^O_2_ generation [93]. Moreover, in the absence of light, salicylic acid can increase the level of ^1^O_2_ through lipid peroxidation [95]. A summary for ^1^O_2_ generation in higher plants is shown in Table 1.

## 3. Singlet Oxygen-Induced Signaling

Singlet oxygen has long been regarded as a cytotoxin because of its short lifetime (0.5–1 µs in biological tissues) and high reactivity [11]. However, more evidence shows that ^1^O_2_ could also act as a multifunctional signal molecule, triggering various stress responses via activating signal transduction cascades [14,17]. The signaling role of ^1^O_2_ was first reported in *C. reinhardtii* in 2001. Leisinger et al. reported that transcripts of *C. reinhardtii Glutathione peroxidase homologous* (*Gpxh*), a thioredoxin-dependent peroxidase catalyzing the reduction of hydrogen peroxide and organic hydroperoxides, was specifically and rapidly induced by ^1^O_2_ produced by photosensitizers including neutral red, methylene blue, and rose bengal [99]. However, most important breakthroughs on ^1^O_2_ signaling came from studies of the Arabidopsis conditional mutants, including but not limited to the *ch1*, *flu*, and *fc2* mutants.

### 3.1. Singlet Oxygen Signaling from Grana Core

Based on the sites of ^1^O_2_ generation, ^1^O_2_-induced signaling in the chloroplast can originate from the grana core (GC, the inner region of the grana stacks) or grana margin (GM, the marginal region of the grana stacks). The *ch1* mutant produces ^1^O_2_ specifically in GC and is thus widely used as a tool to explore ^1^O_2_ signaling originating from GC. The D1 protein in GC could scavenge ^1^O_2_ at the expense of its own oxidation, but there is no evidence to support that the degradation products of D1 act as signal molecules in plants [24]. Using the Arabidopsis *ch1* mutant, Ramel et al. found that ^1^O_2_ produced in GC could oxidize carotenoids, like β-carotene, and produce four volatile derivatives including β-cyclocitral (β-CC), β-ionone (β-I), dihydroactinidiolide (dhA), and α-ionene [100]. Among the four chemicals, β-CC was found to induce the expression of many ^1^O_2_-responsive genes (SORGs), like genes induced by ^1^O_2_ in the *flu* mutant, but had no obvious effect on the expression of H_2_O_2_-responsive genes. Thus, β-CC is considered as a second messenger of ^1^O_2_ in the chloroplast. β-CC-induced gene expression changes were associated with an increased tolerance to photooxidative stress—an external application of β-CC enhances plants’ tolerance to high light stress. In addition, a small number of genes induced by β-CC are also induced by dhA, indicating that β-CC is not the only β-carotenoid-derived messenger involved in the ^1^O_2_ signaling pathway, even though dhA may not be as important as β-CC in mediating chloroplast-to-nucleus retrograde signaling [20,101].

MBS1 is a small zinc finger protein, which was first discovered in the genetic screening of *C. reinhardtii* mutants for early components of ^1^O_2_ signaling. MBS1 is essential for the induction of SORGs. Upon oxidative stress, it accumulates in distinct granules and the cytosol [102]. Function loss of MBS1 leads to deregulation of ^1^O_2_-induced genes after β-CC treatment, indicating that MBS1 is a new downstream intermediate in β-CC signaling pathway that ultimately regulates the photoprotection process of Arabidopsis [103].

In Arabidopsis, a remarkable feature of β-CC-induced transcriptional changes is that it induces various detoxification-related genes, including several GSTs and UDP–glycosyltransferases [100]. The transcriptional cofactor SCARECROW LIKE 14 (SCL14) was proven to play a key role in the retrograde signal transduction triggered by β-CC under photooxidative stress. β-CC binds to SCL14, relocating it to the nucleus and regulating the expression of NAC transcription factors, enhancing the expression of *ANAC002*, *ANAC032*, and *ANAC081*. These transcription factors regulate detoxification-related gene expression, enhancing plant defense [103,104,105]. Meanwhile, β-CC can down-regulate the methyl erythritol-4-phosphate (MEP) pathway in terpenoid biosynthesis, which provides precursors for many important metabolites in photosynthesis, such as chlorophyll, carotenoids, plastoquinone, phylloquinone, and tocopherol, slowing down ^1^O_2_ generation from these tetrapyrroles and providing another layer of protection for plants under strong high light stress [106].

### 3.2. Singlet Oxygen Signaling from the Grana Margin

Unlike that in the GC, ^1^O_2_ generated in GM activates distinct signaling transduction pathways, based mostly on the study of the conditional mutants that generate ^1^O_2_ in GM, i.e., the Arabidopsis *flu* and *fc2* mutant. In the *flu* mutant, excessive photosensitizer Pchlide is accumulated in the dark, allowing ^1^O_2_ generation when plants are transferred to light. Thus, the *flu* mutant provides an excellent system that can produce ^1^O_2_ in a controlled and non-invasive manner [14]. Furthermore, when grown under continuous light, i.e., without Pchlide accumulation and ^1^O_2_ generation, the *flu* mutant shows no visible growth defects, like the wild-type Col-0 plants. After the ^1^O_2_ burst, the mature *flu* plants stop growing immediately, while the seedlings bleach and die. However, all these drastic phenotypic changes are suppressed if *EXECUTER1* (*EX1*) gene is inactivated, even though *flu ex1* mutant generates a similar amount of ^1^O_2_ to its parental *flu* plant upon a dark-to-light shift. In the *flu* mutant, enhanced levels of ^1^O_2_ generated in the thylakoids oxidize the Trp^643^ residue of the EX1 protein, and the subsequent cleavage of the oxidized EX1 protein is necessary for induction of this signaling transduction pathway. The FtsH2 (also called VAR2) protease, localized in the thylakoid of the chloroplast, has been reported in the repair of damaged PSII and turnover of the EX1 protein. In *flu var2*, not only ^1^O_2_-induced cellular responses, but also ^1^O_2_-induced degradation of the EX1 protein is suppressed, underlying that proteolysis of EX1 is crucial for initiating ^1^O_2_-induced and EX1-mediated retrograde signaling [19,107]. Based on genome-wide transcriptome analysis, inactivation of FtsH2 inhibited most (85%) EX1-dependent ^1^O_2_ response genes (SORGs), providing further evidence for the key role of FtsH2 in mediating ^1^O_2_-induced signaling. Although Pchlide is accumulated and ^1^O_2_ is generated non-selectively all over the thylakoid membrane, the ^1^O_2_ sensor EX1 protein is localized in the GM region, making GM an origin of ^1^O_2_ signaling. A combined study of the *flu* and *flu*-related mutants has proved that 1) ^1^O_2_ can also be a highly versatile signal, 2) EX1 is a sensor of ^1^O_2_ signal, and 3) FtsH2-dependent proteolysis of EX1 is necessary for mediating ^1^O_2_ signaling [19,58,108]. A paper published recently reported that the mature EX1 protein relocates from chloroplasts to the nucleus upon ^1^O_2_ release, where it interacts with WRKY (WRKY18 and WRKY40) transcription factors to regulate SORG expression [92]. However, this study is questioned by another study published in 2024 which showed that the mature EX1 protein does not accumulate in the nucleus and does not interact with nuclear WRKY18 or WRKY40 either [109]. Subsequently, a recent study reports that mutation of the *TOC33* gene in the *flu* mutant can suppress largely ^1^O_2_-induced stress responses, even when ^1^O_2_ levels are comparable to those in the *flu* [110]. TOC33 interacts with the UVR domain of EX1 (EX1-UVR) in the chloroplast envelope, promoting an ^1^O_2_-dependent reduction of EX1-UVR in the chloroplasts and an increase in EX1-UVR in the nucleus. The UVR domain in the nucleus interacts with WRKY18 and WRKY40 transcription factors to modulate the expression of SORGs [110].

In addition to EX1, its homolog, EXECUTER2 (EX2), is also involved in mediating ^1^O_2_ signals in Arabidopsis. Function loss of the chloroplastic EX1 protein suppresses largely the up-regulation of SORGs, but does not eliminate these changes. However, inactivation of both EX1 and EX2 led to almost complete inhibition of SORGs [107,111]. Like EX1, EX2 undergoes oxidative post-translational modification at the Trp^530^ of the DUF domain. EX2 localizes in close vicinity to the EX1 protein in the GM region and functions as a negative regulator of the EX1 signalosome through its own ^1^O_2_-dependent oxidation [104,112].

In addition, EX1 and EX2 have been reported to play an important role in plant–pathogen interactions, plastid development, and seed dormancy. During the invasion process, the genus *Alternaria* and other phytopathogenic fungi secrete mycotoxin tenuazonic acid (TeA) that inhibits photosynthesis and causes burst of photosynthetic ^1^O_2_. The functional loss of EX1, or both EX1 and EX2, significantly compromises the expression of ^1^O_2_-responsive nuclear genes and foliar lesions caused by the *Alternaria* pathogens [113]. During the de-etiolation process, EX1 and EX2 perceive and mediate the ^1^O_2_ signal produced in etiolated seedlings, and they orchestrate the expression of photosynthetic gene expression [114]. In addition, EX1 and EX2 also play an important role in seed dormancy and gemination via orchestrating plastid formation [115].

Except for the EX1-dependent pathway, ^1^O_2_ generated on GMs also initiates an EX1-independent pathway, the ^1^O_2_-SAFE1 pathway. SAFE1 was identified in a screening for suppressor mutants in EMS-mutagenized *flu ex1* seeds that restored a *flu*-like phenotype. SAFE1 localizes in the chloroplast stroma, and the release of ^1^O_2_ induces SAFE1 degradation via chloroplast-originated vesicles. Without SAFE1, GMs of chloroplast thylakoids are specifically damaged upon ^1^O_2_ generation [116]. Thus, SAFE1 is recognized as a negative regulator of an ^1^O_2_-induced and EX1-independent pathway, although the ^1^O_2_ sensor of this pathway is still unknown.

**Figure 2:**
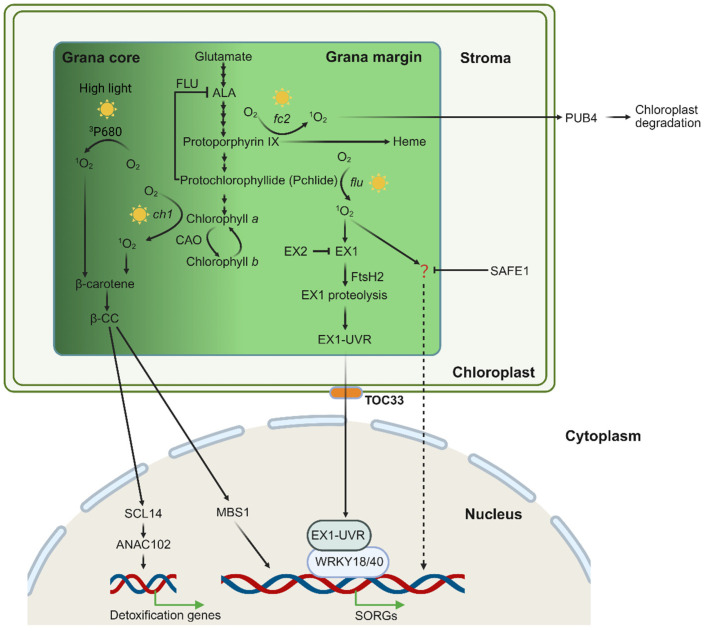
**Scheme of chloroplast ^1^O_2_ generation and signaling in the grana core and grana margin.** Chloroplast ^1^O_2_ is primarily generated during the process of chlorophyll biosynthesis. Under high light, excess energy at PS II leads to the formation of ^1^O_2_ from excited chlorophyll (^3^P_680_ *). This ^1^O_2_ can be quenched by β-carotene, forming β-CC. In the *ch1* mutant, there is ^1^O_2_ generation in the grana core (GC). Excessive ^1^O_2_ oxidizes β-carotene, yielding the volatile signaling molecule β-CC. This compound activates photoprotective gene expression via the zinc finger protein MBS1 and the transcription factor SCL14. The Arabidopsis mutant *fc2* and *flu*, in which the chlorophyll biosynthesis is impaired, accumulates photosensitizing chlorophyll precursors, protoporphyrin IX and Pchlide, respectively. Upon light exposure, these compounds absorb light energy and generate highly reactive ^1^O_2_. The *flu*-produced ^1^O_2_ in the grana margin (GMs) initiates stress-related retrograde signaling (RS) pathways to regulate nuclear gene expression. The sensor protein EX1 detects elevated levels of ^1^O_2_ through oxidation at the W^643^ residue, triggering its hydrolysis by FtsH2. This process releases the UVR domain that is eventually transmitted via the TOC33-dependent mechanism to the nucleus, where it interacts with WRKY transcription factors to regulate the expression of SORGs. The EX2 protein localizes in close vicinity with EX1 and neutralizes ^1^O_2_ via its W^530^ residue oxidation. Additionally, ^1^O_2_ activates an EX1-independent signaling pathway which is suppressed by SAFE1, although the sensor is still uncovered. In the *fc2* mutant, ^1^O_2_ causes chloroplast damage, leading to chloroplast outer envelope ubiquitination by the E3 ligase PUB4 and subsequent vacuolar degradation.

However, the ^1^O_2_-induced signaling pathway in the *fc2* mutant does not rely on the EX1 protein [18]. The *fc2* mutant is defective in plastid ferrochelatases 2 that catalyze the conversion of protoporphyrin IX (Proto IX) to heme in the chloroplast tetrapyrrole biosynthesis pathways [117]. When grown under diurnal light cycles, the *fc2* mutant starts to accumulate Proto IX at dawn and peaks shortly. Proto IX is a strong photosensitizer, generating ^1^O_2_ that leads to chloroplast damage and eventually PCD [18]. However, all these severe phenotypes can be suppressed by mutation of the *PUB4* gene without impairing Proto IX accumulation and the following ^1^O_2_ generation. The *PUB4* gene encodes the Plant U-box 4 (PUB4) E3 ubiquitin ligase that ubiquitinates the outer envelope of the damaged chloroplast, facilitating its degradation in the vacuole [18,118]. Since in the *fc2 pub4* double mutants, much less damaged chloroplasts are observed, PUB4 has been proposed to promote the formation or maturation of damaged chloroplasts. In contrast to *flu*, ^1^O_2_-induced signaling does not rely on the EX1 protein in the *fc2* mutant, in which mutation of the *EX1* gene cannot suppress the ^1^O_2_-induced stress phenotypes [18]. However, whether PUB4 is involved in ^1^O_2_-induced signaling in the *flu* mutant is still unclear. A representative scheme illustrating chloroplast ^1^O_2_ generation and signaling in these mutants is shown in Figure 2.

## 4. Cross Talk Between Phytohormones and ^1^O_2_-Induced Signaling

In addition to photosynthesis, chloroplasts are also major biosynthesis sites of phytohormones such as salicylic acid (SA), jasmonic acid (JA), abscisic acid (ABA), gibberellins, and ethylene [119]. An increased production of ROS in the chloroplast not only directly affects the photosynthetic efficiency, but also affects the biosynthesis and signal transduction of the above hormones [120,121,122].

### 4.1. Salicylic Acid and ^1^O_2_ Signaling

SA is a key phytohormone that regulates diverse signaling pathways, particularly plant immune responses [123]. In plants, SA is synthesized through two distinct pathways—the ICS pathway and the PAL pathway, both originating from chorismate in the chloroplast.

In the ICS pathway, chorismate is first isomerized to isochorismate by isochorismate synthase (ICS) enzymes. Isochorismate is then transported into the cytosol via the EDS5 transporter located on the chloroplast envelope. There, it is conjugated with L-glutamate by a cytosolic amidotransferase, forming isochorismate-9-glutamate (ICS-Glu), which spontaneously decomposes into SA and 2-hydroxy-acryloyl-N-glutamate. In the PAL pathway, chorismate is converted into phenylalanine within plastids. Phenylalanine is subsequently exported to the cytosol and deaminated to trans-cinnamic acid (t-CA) by phenylalanine ammonia-lyase (PAL). t-CA is further metabolized into SA via intermediates such as ortho-coumaric acid and benzaldehyde [124,125,126].

Under high light stress, β-CC generated in the PSII RC promotes SA biosynthesis by upregulating ICS1 expression in Arabidopsis [127]. This β-CC-induced SA accumulation, along with associated changes in nuclear gene expression, enhances plant tolerance to excessive light. During this acclimation process, EDS1 and NPR1 (Nonexpressor of Pathogenesis-Related Genes 1), a central component of SA signaling, positively regulate SA biosynthesis and signaling, respectively [127]. In the cytoplasm, NPR1 typically forms oligomers stabilized by disulfide bonds between cysteine residues in its monomers [128,129]. SA accumulation alters the cellular redox state, reducing these disulfide bonds and liberating NPR1 monomers, which then translocate into the nucleus. There, NPR1 interacts with TGA transcription factors to activate defense-related gene expression [128,130].

In the *flu* mutant, SA-responsive genes such as *EDS1*, *PATHOGENESIS RELATED1* (*PR1*), and *PR5* are strongly induced upon ^1^O_2_ release [131]. Inactivation of *EDS1* in the *flu* mutant does not affect ^1^O_2_-induced oxylipin generation, growth inhibition, and the initiation of PCD, but it does allow plants to recover much faster from ^1^O_2_-induced growth inhibition and it also suppresses the spread of necrotic lesions in leaves [131]. It is proposed that ^1^O_2_ activates a complex stress-response program with EDS1 playing a key role in initiating and modulating several steps of it. This program includes not only responses to oxidative stress, but also responses known to be activated during plant–pathogen interactions and wounding [131]. Moreover, a chloroplast-localized protein, named the calcium-sensing receptor (CAS), has been shown to act upstream in ^1^O_2_-triggered retrograde signaling, influencing plant immunity and SA biosynthesis [132]. It was shown that pathogen-associated molecular pattern (PAMP) signals are quickly relayed to chloroplasts and evoke CAS-dependent transient Ca^2+^ signals in the chloroplast that are responsible for both the PAMP-induced basal resistance and R gene-mediated hypersensitive cell death. Transcriptome analysis demonstrates that CAS-mediated transcriptional reprogramming is achieved via chloroplast ^1^O_2_-mediated retrograde signaling [132].

**Figure 3:**
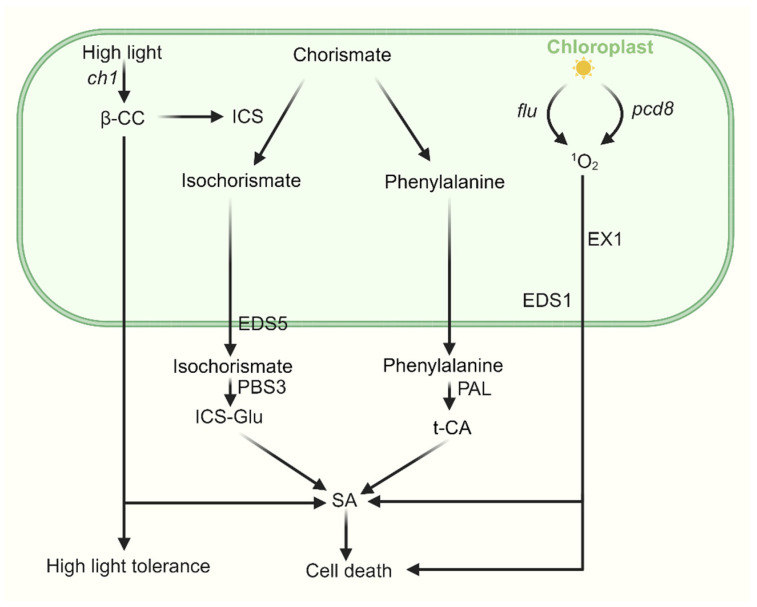
**The cross talk between SA and ^1^O_2_-induced signaling.** SA is synthesized through two distinct pathways—the ICS pathway and the PAL pathway—both originating from chorismate in the chloroplast, with final biosynthesis occurring in the cytosol. Under high light stress, β-CC enhances plant tolerance by upregulating *ICS1* expression and promoting SA biosynthesis, accompanied by associated changes in nuclear gene expression. In *pcd8* RNAi mutants, the accumulation of ^1^O_2_ stimulates biosynthesis of SA in an EX1-dependent manner. In the *flu* mutant, upon ^1^O_2_ release, SA-responsive genes are strongly induced, activating a complex stress-response program in which *EDS1* plays a key role.

Recent studies suggest that GUN1-mediated retrograde signaling also modulates the expression of defense-related genes associated with SA signaling [133,134]. GUN1 signaling transcriptionally represses GOLDEN2-LIKEs (GLKs), key regulators of chloroplast development [135]. GLKs are involved in SA signaling, and both SA and the SA-induced protein SIGMA FACTOR BINDING PROTEIN 1 (SIB1) can directly influence GLK activity [136]. Prolonged SA exposure negatively affects tetrapyrrole biosynthesis, impairing chloroplast development. Notably, this SA-induced defect in chloroplast biogenesis is more pronounced in *gun1* mutants, implying that GUN1-mediated retrograde signaling may protect chloroplast development by suppressing GLK-driven defense responses linked to SA [133]. These findings point to a likely interconnection between chloroplast-derived reactive oxygen species (ROS) and SA-mediated signaling. Recently, a study reported that in dark conditions, salicylic acid-mediated lipid peroxidation leads to elevated levels of ^1^O_2_, which subsequently trigger EX1-dependent retrograde signaling. Loss of *EX1* function partially alleviates the SA-induced hypocotyl growth inhibition in the *npr1* mutant. This mechanism may involve the reactivation of *SAUR* gene expression at the transcriptional level in the *npr1* genetic background [95]. Although the roles of chloroplast ROS and SA in plant stress responses are well established, the molecular and genetic interactions between these components, as well as the potential function of SA in retrograde signaling, remain incompletely understood. A simplified model for the relationship of ^1^O_2_ and JA is shown in Figure 3.

### 4.2. Jasmonate and ^1^O_2_ Signaling

Jasmonates (JAs) are lipid-derived cyclopentanones that include JA and its various derivatives. As the major immunity hormone, the jasmonate family of oxylipins promote plant defense to necrotrophic pathogens, chewing insects, and mechanical wounding. Within chloroplasts, PHOSPHOLIPASE A1 (PLDA1) acts on membrane lipids to release α-linolenic acid, which is subsequently oxidized and cyclized by 13-LIPOXYGENASE (13-LOX), ALLENE OXIDE SYNTHASE (AOS), and ALLENE OXIDE CYCLASE (AOC) to form the JA precursor 12-oxo-phytodienoic acid (OPDA) [137,138,139]. OPDA is then transported via the channel formed by the JASSY protein on the chloroplast envelope to peroxisomes [140], where it is catalyzed to form JA by OPDA REDUCTASE 3 (OPR3), ACYL-CoA OXIDASE (ACX), MULTIFUNCTIONAL PROTEIN (MFP), and L-3-KETOACYL CoA THIOLASE (KAT) [139].

Studies on the *flu* mutant have shown that, when re-exposed to light after dark adaptation, it accumulates OPDA and JA in an EXECUTER 1 (EX1)-dependent manner [141]. When the *flu* mutant was crossed with the JA-depleted *opr3* mutant, the cell death phenotype in the resulting *flu opr3* was largely suppressed, indicating that JA promotes ^1^O_2_-induced cell death in *flu*. Whereas other oxylipins such as OPDA and dnOPDA antagonize this cell death-inducing activity of JA [142]. Similarly, during the invision process of necrotrophic *Alternaria* fungal pathogens on plant species, genes implicated in JA biosynthesis and signaling are induced rapidly, and mutation of the *EX1* gene compromises the necrotrophic disease in Arabidopsis. Moreover, the foliar lesion formation caused by *Alternaria* pathogen in JA-deficient mutants *lox3*, *dde2*, *aoc3,* and *jar1* is much smaller than that in wild-type controls, indicating that JA promotes *Alternaria* pathogen-induced cell death and disease development [113].

Interestingly, under moderate high light stress that does not induce cell death, β-CC-mediated acclimation does not influence JA biosynthesis or signaling. However, under severe high light stress that eventually leads to cell death, JA biosynthesis genes, such as *LOX2*, *AOC1*, *AOS*, and *OPR1,* are upregulated [16,100,143,144]. When *ch1* mutants were exposed to intense high light (1400 μmol m^−2^ s^−1^), the expression of *LOX2* and *LOX3* was induced, JA accumulated, and cell death occurred. Conversely, when *ch1* mutants were first acclimated (short exposure to medium light intensity), JA biosynthesis and cell death were suppressed under the same high light conditions [17]. Moreover, *ch1 aos* double mutants exhibited enhanced tolerance to photooxidative stress under high light [17]. These findings indicate a potential interaction between β-CC and JA biosynthesis/signaling under extreme high light, where JA levels influence both programmed cell death (PCD) and acclimation. Under milder stress, β-CC-mediated retrograde signaling promotes acclimation and suppresses JA biosynthesis.

**Figure 4:**
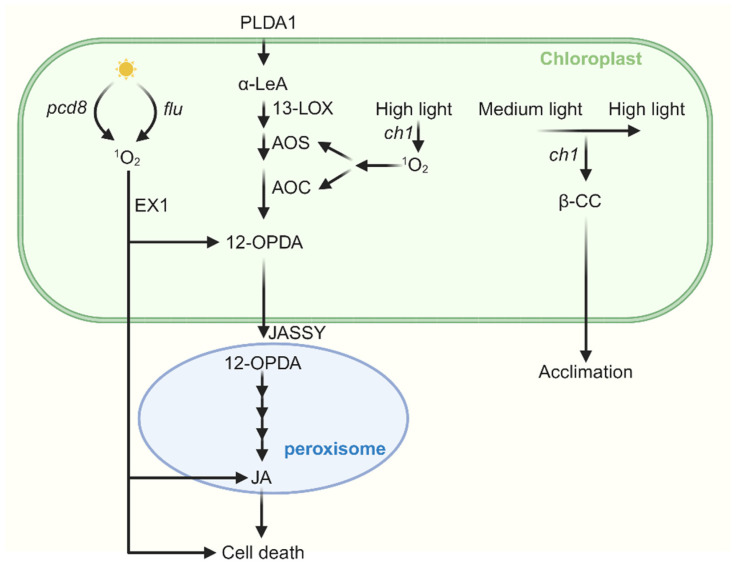
**The cross talk between JA and ^1^O_2_-induced signaling.** Jasmonic acid (JA) synthesis begins with α-LeA, which is converted to 12-OPDA by 13-LOX, AOS, and AOC. 12-OPDA is then transported from the chloroplast via the outer envelope protein JASSY into peroxisomes, where it is further catalyzed to JA. In the *flu* mutant, when dark-adapted plants are re-exposed to light, the accumulation of both OPDA and JA occurs in an EX1-dependent manner. Furthermore, JA promotes ^1^O_2_-induced cell death in *flu*. In *pcd8* RNAi mutants, the accumulation of ^1^O_2_ stimulates biosynthesis of JA and cell death. Under severe high light stress, JA biosynthesis genes are upregulated in the *ch1* mutant, leading to JA accumulation and subsequent cell death. Interestingly, when *ch1* mutants are first acclimated to medium light and then transferred to the same high light conditions, both JA biosynthesis and cell death are suppressed.

The gene *OXI1*, which encodes an AGC kinase family protein localized at the cell periphery, is strongly upregulated in *ch1* mutants under cell death-inducing high light [145]. The *ch1 oxi1* double mutants showed a coordinated reduction in JA levels and PCD, resulting in a marked suppression of both photooxidative damage and necrosis phenotypes [145]. Conversely, overexpression of *OXI1* increased the JA levels and sensitivity to high light stress. Exogenous JA application restored photooxidative damage and cell death in *ch1 oxi1* mutants, indicating that *OXI1* regulates ^1^O_2_-induced PCD via JA. Further studies revealed that DEFENDER AGAINST CELL DEATH 1 (DAD1) and DAD2, two negative regulators of PCD, modulate *OXI1* expression under high light [146]. Overexpression of *DAD1* and *DAD2* suppressed *OXI1* expression and reduced JA levels, thereby decreasing sensitivity to photooxidative damage. The relationship of ^1^O_2_ and JA is simply summarized in Figure 4.

Notably, JA treatment upregulated the SA biosynthesis gene, leading to more severe leaf damage—a phenomenon absent in the SA-deficient mutant *ics1*, indicating that SA acts downstream of JA in promoting PCD [146]. Additionally, exogenous SA treatment enhanced *DAD* expression while suppressing *OXI1*. These results suggest that DADs and *OXI1* antagonistically regulate PCD through the modulation of JA and SA pathways. Overall, the relationships among JA, SA, and various retrograde signaling pathways are complex and need further investigation to fully elucidate their interactions.

## 5. Singlet Oxygen Induces Chloroplast Changes Under Stress

Under ideal environmental conditions, plant cells generate ROS at a low, steady rate, maintaining their concentration within a safe threshold. However, under stress conditions such as high light, high temperatures, drought, or salinity, the balance of photosynthesis is disrupted. This leads to over-reduction in electron transport chains and a consequent surge in ROS production. These highly reactive molecules inflict damage on essential cellular components—including lipids, carbohydrates, proteins, enzymes, and DNA—through oxidative degradation. Such cumulative harm disrupts molecular and cellular integrity, ultimately resulting in plant mortality [147,148].

Chloroplasts are sensitive to stress. Stress affects the structure and components of the thylakoid membrane. A significant transcriptomic correlation has been observed across multiple abiotic stress conditions and ^1^O_2_ accumulation [94]. These stresses—including drought, salinity, mechanical injury, and high light exposure—were commonly linked to ^1^O_2_-induced disruptions in mRNA translation. This effect is mediated through the direct oxidation of RNA by singlet oxygen [149].

### 5.1. Abiotic Stress

#### 5.1.1. High Light

High light can induce the unstacking of thylakoids and randomization of pigment-protein complexes of chloroplasts [150]. The highly photosensitive mutant *ch1* has less chlorophyll *b* and releases ^1^O_2_ in PSII under photooxidation stress [17]. Light stress damages PSI and PSII, decreases photosynthesis rate, and leads to photoinhibition and photooxidative stress [151]. PSII is one of the primary targets for damage induced by visible and ultraviolet light. High light and UVB can damage the PSII complex at Mn clusters, and induces modification or loss in the function of the Q_A_ and Q_B_ quinone electron acceptors [152,153]. However, there are some differences between high light stress and UVB stress. Under high light stress, the excessive light energy absorbed by chlorophyll and carotenoids can destroy the integrity of the PSII reaction center in the absence of a functional OEC (oxygen-evolving complex) [154]. Under ultraviolet light, the energy absorbed by the OEC leads to changes in the valency of Mn, which in turn can oxidize other components of PSII [155]. When plants are exposed to more light energy than they need, chlorophyll molecules become over-excited and generate an excess of excitation energy. The over-excitation of PSII promotes the formation of triplet-state excited chlorophyll, which can then excite oxygen to produce ^1^O_2_ [2,44].

#### 5.1.2. High Temperature

High temperatures, high salinity, and drought stress do not directly damage PSII as high light stress does. However, they can affect the content of photosynthetic pigments and the repair of PSII [156]. In extreme temperatures, high salinity stress can inhibit the de novo synthesis of the D1 protein, and affect the phosphorylation dynamics within the PSII complex [157,158]. This process is crucial for initiating the degradation and reassembly of PSII [151]. Moreover, abiotic stresses (e.g., extreme temperatures and high salinity) can affect the grana stacking and thylakoid membrane heterogeneity, which in turn impacts the repair of PSII [159]. When exposed to heat stress, the thylakoid membranes have lower thermostability compared to the chloroplast membranes, and the membrane potential is disrupted [160]. High temperature stress induces significant changes in the structure of thylakoid membranes in plants, accompanied with an increase in peroxidation of thylakoid lipids [161,162]. Moderate heat stress does not damage PSII, but it reduces the photosynthetic rate and increases thylakoid leakiness. When plants were exposed to increasing temperature, researchers observed a dissociation of the light-harvesting units of Photosystem II, and phase-separated deaggregates of non-bilayer-forming lipids which can be restored under recovery conditions [163,164]. In addition, high temperatures inactivate Rubisco, thereby affecting carbon fixation [165]. When plants are subjected to low temperature and high light stress, species such as the grasses *Sorghum, Paspalum,* and the legume soybean, representing the C4 and C3 photosynthetic pathways, exhibit a reduction in the size of starch grains within their chloroplasts [166]. Extreme temperatures cause proteins to precipitate from the thylakoid membranes, affecting photosynthetic phosphorylation, leading to partial inactivation of electron transport through Photosystem II, and the types of precipitated proteins are related to osmolarities [161,167].

#### 5.1.3. Drought and Salt Stress

Drought stress imposes multifaceted constraints on photosynthesis by affecting chloroplast development, reducing Chl content, damaging photosynthetic thylakoid membranes, limiting CO_2_ availability through stomatal closure, altering enzyme activities, changing the membrane lipid contents, and inducing oxidative stresses. When etiolated wheat seedlings are subjected to drought stress simulated by polyethylene glycol and then exposed to light, the number of chloroplasts formed is significantly reduced, and the number of grana thylakoids within the chloroplasts decrease [168]. Meanwhile, Chl production was significantly decreased in drought-stressed leaves, and swelling or dilation of thylakoid membranes become common during the subsequent greening process [168]. Under osmotic stress, the integrity of the chloroplast envelope remains unaffected, but the level of ATP synthesis is impacted, with a partial inactivation of electron transport through Photosystem II occurring. Drought stress affects the content of membrane lipids. When winter wheat encounters water stress, phosphatidylcholine (PC), phosphatidylethanolamine (PE), and phosphatidylglycerol (PG) are the main targets for degradation, with PC being particularly affected, while the molecular species of monogalactosyldiacylglycerol (MGDG) and digalactosyldiacylglycerol (DGDG) exhibit different degradation time courses [169]. Moreover, short-term drought stress triggered a rapid disassembly of the light-harvesting complex II (LHCII), the amount of the PSII-LHCII supercomplexes and LHCII assemblies are markedly reduced [170,171].

Different levels of salt stress have varying effects on plants, for instance, 50 mM NaCl treatment on *Robinia pseudoacacia* increases antioxidant enzyme activity and upregulates the expression of the ion transport-related genes and chloroplast development-related genes, while severe salt stress has the opposite effect [172]. Furthermore, the high salt stress also reduced the photochemical efficiency of PSI and PSII and decreased the supercomplexes such as PSII-LHCII in pea (*Pisum sativum*) [173].

#### 5.1.4. Heavy Metal

Heavy metal toxicity can also damage the chloroplasts and photosynthetic systems of plants. When *Oryza sativa* was treated with high concentrations of copper (II) oxide nanoparticles (CuO NPs), researchers observed the accumulation of CuO NPs in chloroplasts and a lower number of thylakoids per granum [174]. Chromium (Cr) and lead (Pb) cause distortion of the thylakoid, distortion of chloroplast membrane, and changes in the chloroplast structure [175]. A summary of ^1^O_2_-induced chloroplast and photosystem changes under abiotic stresses is shown in Table 2.

### 5.2. Biotic Stress

Chloroplasts are among the most dynamic organelles in plant cells. Beyond their central role in photosynthesis and the synthesis of key plant hormones, they also actively contribute to defense signaling [176]. As a result, chloroplasts have become a critical target for numerous pathogens [177]. Both viral and bacterial invaders employ a variety of sophisticated strategies to disrupt chloroplast structure and function, thereby compromising plant immunity and facilitating their own colonization [176].

A previous study has reported that the infection of *Eclipta yellow vein virus* (EcYVV-IN) on *Andrographis paniculata,* resulting in the decrease in carotenoid content and ^1^O_2_ quenching, highlighted the alteration in redox status caused by virus-induced biotic stress on the plants [178]. Cercosporin, a toxin secreted by the necrotrophic fungus *Cercospora*, induces the expression of ^1^O_2_-responsive genes. This activation promotes programmed cell death and foliar tissue damage in host plants, ultimately enhancing fungal colonization [179,180]. Tenuazonic acid (TeA), produced by the genus *Alternaria* and other phytopathogenic fungi, binds to D1 proteins of the PSII reaction center and leads to ^1^O_2_ burst that is implicated in chloroplast damage and chloroplast-to-nucleus retrograde signaling.

In plant cells, one of the most significant challenges faced by viruses is the silencing of their RNA [181]. To evade this defense, viruses compartmentalize their replication within specialized vesicles and chloroplasts—structures believed to lack RNA silencing machinery [182,183]. Viral proteins directly target chloroplasts or interact with chloroplast-associated proteins, leading to structural and functional alterations within the organelle. These disruptions commonly manifest as visible symptoms in infected leaves, such as chlorosis, mottling, or mosaic patterning [184]. Virus infection can also inhibit the photosynthetic function of chloroplasts and activate genes related to defense response (genes related to JA and SA signaling pathway) [185,186].

Calcium and ROS serve as the initial signaling molecules in chloroplast-mediated immunity. Following the recognition of pathogen-associated molecular patterns (PAMPs) by pattern recognition receptors (PRRs), rapid phosphorylation cascades are triggered alongside H_2_O_2_ production, leading to a rise in cytoplasmic calcium ion (Ca^2+^) levels [187,188]. This calcium signal subsequently activates the thylakoid-localized calcium-sensing receptor (CAS), resulting in increased Ca^2+^ concentration within the chloroplast stroma [189]. Concurrently, H_2_O_2_ may potentiate early immune responses originating in the chloroplast [190]. The activation of pattern-triggered immunity (PTI) induces a robust burst of chloroplast-derived reactive oxygen species, which in turn stimulates the expression of defense-related genes and represses nuclear-encoded chloroplast genes [188]. This coordinated response ultimately attenuates photosynthetic activity and impairs plastid development. CAS contributes to both basal resistances triggered by PAMPs and hypersensitive cell death mediated by resistance (R) genes. Functioning upstream of SA accumulation, CAS is implicated in the activation of PAMP-induced defense-related genes and the suppression of chloroplast-encoded genes via ^1^O_2_-mediated retrograde signaling. This mechanism facilitates chloroplast-driven transcriptional reprogramming during the plant immune response [122,132].

In recent years, a plastid (chloroplast) extension structure named stromule has garnered increasing attention as an emerging focus in plant cell biology. These highly versatile structures extend from plastids and form intimate connections with the nucleus and other organelles, facilitating inter-organellar communication and material exchange [191]. In the plant immune response process, chloroplast-derived stromules play a role in promoting the movement of chloroplasts to the perinuclear region, transducing and amplifying pro-defense signals originating from chloroplasts, and participating in the induction of programmed cell death [192,193]. The formation of stromules during plant immunity is a multifaceted process that involves a complex interplay of signaling components. Initially, plants recognize effectors produced by pathogens through immune receptors, triggering Effector-Triggered Immunity (ETI). Then, stromules can emerge from the surface of chloroplasts as tubular structures, extend along microtubules, and anchor to actin filaments around the nucleus to promote perinuclear chloroplast clustering [194,195]. Stromules often form in response to abiotic and biotic stress, and their formation is associated with ROS, particularly H_2_O_2_ [196]. Caplan et al. found that stromules induction is a general response initiated by viral effectors such as the p50 helicase domain of TMV replicases and bacterial effectors such as AvrBS2 during the early phases of a hypersensitive response (HR-PCD). Moreover, stromule induction was not limited to the initially affected cells but also occurs in intact neighboring cells [196].

## 6. Conclusions Remarks

As a by-product of photosynthesis, ^1^O_2_ has been recognized from a simple toxic molecule into a central node within a complex signaling network. This review systematically elaborates on the generation of ^1^O_2_ in plant cells and elucidates the molecular mechanisms underlying its perception and downstream responses. More importantly, there exists an intricate cross talk between ^1^O_2_ and the signaling networks of two key defense hormones—JA and SA. Firstly, ^1^O_2_ production is often linked to photosynthetic disruption and early stress responses, which can activate JA biosynthesis and signaling. This interaction enables plants to initiate defense programs against herbivorous insects and pathogens. Secondly, ^1^O_2_ and SA signaling exhibit a complex relationship marked by both antagonism and coordinates. Under high light stress, for instance, ^1^O_2_ promotes SA accumulation and enhances plant tolerance. Conversely, ^1^O_2_ generated during the transition from dark to light can also induce SA accumulation and trigger cell death. This dynamic cross talk allows plants to adjust their defense strategies appropriately in response to varying ^1^O_2_ levels under stress conditions.

Despite significant progress, several key questions in this field remain to be explored. The precise intersection of ^1^O_2_ with JA/SA pathways is still not fully understood. Identifying “bridge proteins” that sense the redox state of ^1^O_2_ and regulate JA/SA signaling will be crucial for exploring the molecular basis of these interactions. Future studies should focus on identifying key components of ^1^O_2_ sensing and signaling, elucidating how ^1^O_2_ signaling integrates with JA/SA networks under combined stresses—to more accurately reflect plant survival strategies in natural environments, as well as exploring the usage of ^1^O_2_ in modern agriculture and medical fields. In summary, ^1^O_2_ constitutes a sophisticated signaling system that links photosynthesis, oxidative stress, and hormone-mediated defense networks. Further research into its production, transduction, and cross talk with phytohormones will not only deepen our understanding on plant environmental adaptation and immune balance but also provide novel theoretical insights and technical approaches for addressing agricultural challenges in the context of global climate change.

## Figures and Tables

**Table 1:** A summary of singlet oxygen generation in plant.

Classification	Source	Core Mechanism	Related Mutant
Photosynthesis-related	The light-harvesting antenna complex (LHC)	The triplet excited state ^3^Chl * transfers energy to ^3^O_2_ to generate ^1^O_2_	*ch1* under high light
	Photosystem II reaction center (PSII RC)	Under high light, ^1^P_680_ * decays into ^3^P_680_ *, resulting in ^1^O_2_ generation	Not applicable
	Plastoquinone (PQ) pool	Besides electron mediator, PQ is also an ^1^O_2_ scavenger	*abc1k1* under red or white light
Chlorophyll biosynthesis intermediates	Intermediates of tetrapyrrole synthesis (e.g., Pchlide, Proto IX, Uro III)	Defective in negative regulator or key enzymes of tetrapyrrole biosynthesis, accumulation of photosynthesizing products	*flu* after DL transfer; *fc2* after DL transfer; *pcd8* after DL transfer
Chlorophyll degradation products	Intermediates chlorophyll degradation (e.g., Pheophorbide *a* and RCC)	The absence of PAO or RCCR enzyme activity leads to accumulation of photosynthesizing chlorophyll degradation intermediates	*acd1* and *lls1* (maize) accumulates Pheophorbide *a*; *acd2* accumulates RCC
Seedling de-etiolation	Pchlide of the PLB in etiolated plastid	*pifs* mutant accumulates Pchlide in the dark and generates ^1^O_2_ after illumination	*pif1*, *pif3* and *pif5* mutant grown under DL condition
Environmental stress	Biological/abiotic stress activates multiple pathways and induces non-photosynthetic-dependent ^1^O_2_ production in plant tissues	Leaf damage induces lipoxygenase mediated-LPO and produces ^1^O_2_	*lox2* produces less ^1^O_2_ after leaf damages
		Drought changes the osmotic potential and produces ^1^O_2_ in root	Not applicable
		Cold and high light stress induce ^1^O_2_ production in leaf	Not applicable

**Table 2:** Responses of plant chloroplasts and photosynthetic systems to different abiotic stresses.

Stress	Main Targets	Key Changes
High light	PSII; PSI; Thylakoids; OEC	Thylakoid unstacking; Pigment-protein complex disorganization; Oxidation of PSII; Mn-O valence broken in OEC
High temperature	PSII repair; Thylakoid; Rubisco; Lipids	Inhibition of PSII degradation and reassembly; Inhibition of de novo synthesis of D1 protein and altering PSII phosphorylation; Inhibition of Rubisco activity and carbon fixation; Reduced thylakoid membrane thermostability and disrupted membrane potential; Dissociation of PSII LHC and phase separation of non-bilayer lipids; Increased thylakoid lipid peroxidation
Drought	Pigments; Thylakoids; Lipids; PSII-LHCII complexes PSII; PSI	Decreased chlorophyll content; Degradation of membrane lipids (PC, PE, PG as major targets); Swelling and vesiculation of thylakoid membranes; Reduced photochemical efficiency of PSI and PSII; Inhibition of ATP synthesis and inactivation of PSII electron transport
Heavy metal	Thylakoids; Envelope; Photosynthetic apparatus	Distortion of thylakoids and envelope (e.g., Cr, Pb). Reduction in thylakoid number per granum (e.g., CuO); Direct damage to chloroplast membranes and disruption of photosynthetic apparatus assembly

## Data Availability

No new data were created or analyzed in this study.

## Abbreviations

ABA, Abscisic acid; ACX, Acyl-CoA oxidase; ALA, δ-aminolevulinic acid; AOC, Allene oxide cyclase; AOS, Allene oxide synthase; CAO, Chlorophyll *a* oxygenase; Car, β-carotenes; CAS, Calcium-sensing receptor; *ch1*, *chlorina1*; Chl, Chlorophyll; Chl *a*, Chlorophyll *a*; Chl *b*, Chlorophyll *b*; ^1^Chl *, Singlet excited state of chlorophyll; ^3^Chl *, Triplet excitation state of chlorophyll; Chlide, Chlorophyllide; DAD1, *Defender against cell death 1*; DGDG, Digalactosyldiacylglycerol; dhA, Dihydroactinidiolide; EcYVV-IN, *Eclipta yellow vein virus*-[IN]; ETI, Effector-Triggered Immunity; EX1, EXECUTER1; FeCh, Ferrochelatase; GC, Grana core; GLKs, *Golden 2-likes*; Glu-TR, Glutamyl-tRNA reductase; GM, Grana margin; *Gpxh*, *C. reinhardtii Glutathione peroxidase homologous*; H_2_O_2_, Hydrogen peroxide; ICS, Isochorismate synthase; ICS-Glu, Isochorismate-9-glutamate; JA, Jasmonic acid; KAT, L-3-ketoacyl CoA thiolase; LHC, Light-harvesting antenna complex; MEP, Methyl erythritol-4-phosphate; MFP, Multifunctional protein; MgCh, Magnesium chelatase; MgCY, Mg-Proto IX monomethylester cyclase; MGDG, Monogalactosyldiacylglycerol; MgMT, Mg-Proto IX methyltransferase; MgP, Mg–protoporphyrin IX; NPQ, Non-photochemical quenching; NPR1, Nonexpressor of pathogenesis-related genes 1; ^1^O_2_, Singlet oxygen; ^3^O_2_, Triplet molecular oxygen; $O_{2}^{\cdot -}$, Superoxide radical; OH^•^, Hydroxyl radical; OPDA, 12-Oxo-phytodienoic acid, OPR3, OPDA reductase 3; PAL, Phenylalanine ammonia-lyase; PAMP, Pathogen-associated molecular pattern; PAO, Phenylalanine oxygenase; PC, Phosphatidylcholine; PCD8, Programmed cell death 8; Pchlide, Protochlorophyllide *a*; PE, Phosphatidylethanolamine; pFCC, primary fluorescence Chl catabolites; PG, Phosphatidylglycerol; Pheo, Pheophytin; PLB, Prolamellar body; PLDA1, Phospholipase A1; POR, Pchlide oxidoreductase; PQ, Plastoquinone; PR1, Pathogenesis related 1; Proto IX, Protoporphyrin IX; PRRs, Pattern recognition receptors; PTI, Pattern-triggered immunity; PUB4, Plant U-box 4; Q_A_, Quinone A; RC, PSII reaction center; RCC, Red chlorophyll catabolite; RCCR, RCC reductase; ROS, Reactive oxygen species; SA, Salicylic acid; SCL14, Scarecrow like 14; SIB1, Sigma factor binding protein 1; SORGs, ^1^O_2_-responsive genes; SOSG, Singlet oxygen sensor green; t-CA, trans-cinnamic acid; TeA, Tenuazonic acid; Uro III, Uroporphyrinogen III; β-CC, β-cyclocitral; β-I, β-ionone; 13-LOX, 13-Lipoxygenase.
